# Papillary syringocystadenoma in an uncommon location^☆^^☆☆^

**DOI:** 10.1016/j.abd.2019.02.011

**Published:** 2019-12-18

**Authors:** Giovanna de Araujo Horcel, Juliana Milhomem, Samuel Henrique Mandelbaum, Rodrigo Ieiri

**Affiliations:** Dermatology Service, Santa Casa de São José dos Campos, São José dos Campos, São Paulo, SP, Brazil

Dear Editor,

Papillary syringocystadenoma is a rare neoplasm of sweat glands, which is present at birth in 50% of cases.^1^ It has predominantly apocrine differentiation, although an eccrine origin has been described in some reports.^2^, ^3^

It usually presents as papule or plaque with a crustal surface that occurs almost exclusively in the head and cervical region.^4^

This report details the clinical observations as well as the dermatoscopic and histopathological findings of a case in an unusual location.

A 6-year-old male, a native and resident of São José dos Campos, SP, presented a lesion with progressive growth five years ago on the left flank. At the dermatological examination, a papule of pink-erythematous coloration was observed, with a smooth surface and a fibroelastic consistency, measuring 5 mm × 3 mm (Fig. 1). Dermoscopic examination showed rounded structures of whitish-yellow color separated by whitish linear structures on an erythematous background (Fig. 2). The patient was referred for excision. Histopathological examination revealed the following: cystic invaginations covered by cells, sometimes squamous and sometimes columnar, with papilliferous projections to the light; tubular glands with large lights, covered by apocrine cells (Fig. 3).

**Figure 1:**
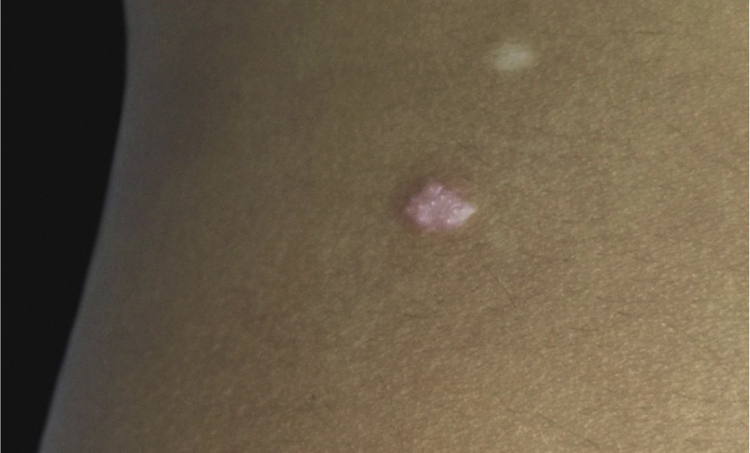
Rose-colored papule on the left flank.

**Figure 2:**
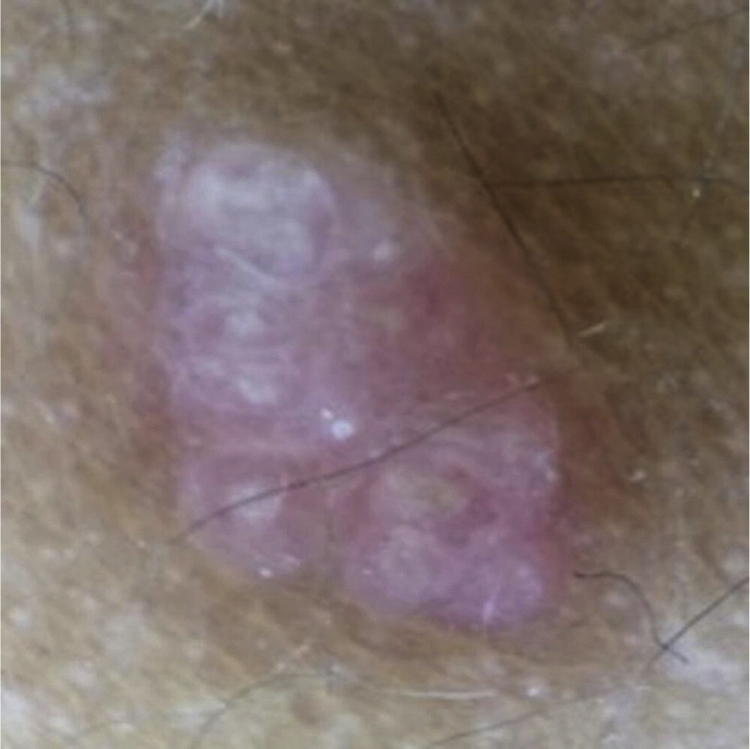
Dermoscopic examination showing rounded yellowish-whitish structures separated by linear whitish structures on an erythematous background.

**Figure 3:**
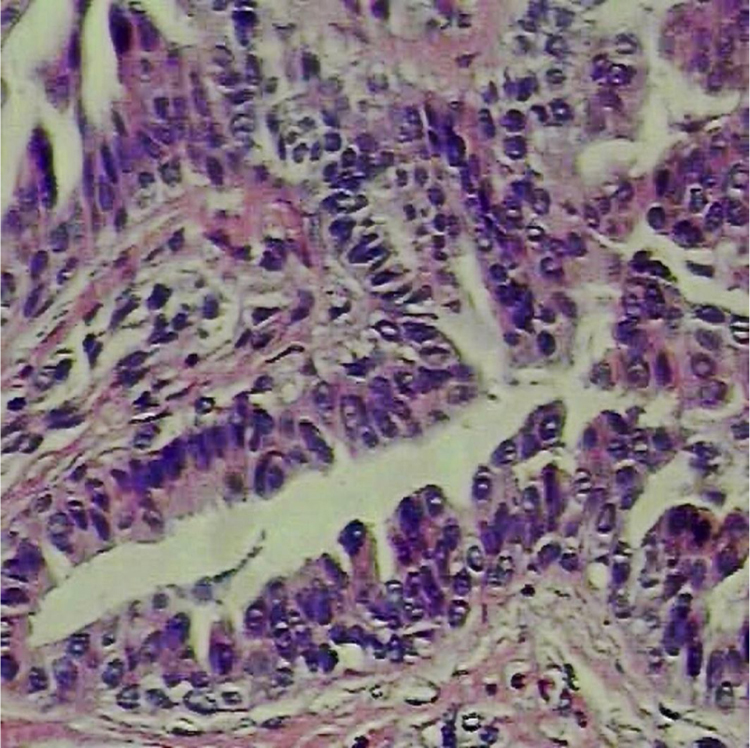
Histopathological examination: cystic cavity coated by basal cuboidal cells and columnar apocrine cells (Hematoxylin & eosin, ×400).

Papillary syringocystadenoma (SCAP) is a rare adnexal tumor most often derived from apocrine cells.^3^, ^4^ It predominates in children and adolescents, and is observed at birth in 50% of cases,^1^ which differs from the case reported above.

In 75% of cases, it is located on the head or cervical region. Some cases in other topographies have already been described (scrotal region, vulva, back, abdomen, and axilla). These locations, as well as that of the case described, are even rarer. When located on the scalp, it may be associated with the sebaceous nevus of Jadassohn.^4^

Despite variable clinical presentation, a papule plaque is the most commonly found lesion type. In the majority, it is asymptomatic, but it can present pruritus, pain, and/or bleeding; it usually presents progressive growth, as in the case described.^1^, ^4^

To date, articles on the dermoscopic findings of SCAP are scarce. There is a description of a horseshoe vascular pattern, which was not observed in the present case.^3^, ^5^

SCAP can infect, bleed, ulcerate, and, in rare cases, it can progress to basal cell carcinoma (9%) or papillary syringo-cystadenocarcinoma. For these reasons, it was decided to perform the excision.^5^

## Financial support

None declared.

## Authors’ contribution

Giovanna de Araujo Horcel: Conception and planning of the study; composition of the manuscript.

Juliana Milhomem: Conception and planning of the study.

Samuel Henrique Mandelbaum: Critical review of the literature; critical review of the manuscript.

Rodrigo Ieiri: Participation in the design of the study.

## Conflicts of interest

None declared.
